# Training Protocol for Nellore Cattle in Respirometry Flow Trials Using Non-Ventilated Facial Mask

**DOI:** 10.3390/ani14192888

**Published:** 2024-10-08

**Authors:** Érika Cristina Dias de Oliveira Brelaz, Gustavo André Bernado Moura, Vinícius de França Carvalho Fonsêca, Juliete Amanda Theodora de Almeida, Bruno Rodrigo Simão, Alex Sandro Campos Maia

**Affiliations:** 1 Innovation and Sustainability in Animal Biometeorology InsBio, School of Agricultural and Veterinary Sciences, São Paulo State University (UNESP), Jaboticabal 14884-900, Brazil; gustavogto72@gmail.com (G.A.B.M.); vinicius_fonseca86@hotmail.com (V.d.F.C.F.); juliete.almeida@unesp.br (J.A.T.d.A.); bruno@ufersa.edu.br (B.R.S.); alex.maia@unesp.br (A.S.C.M.); 2 Federal Institute of Education, Science and Technology of Amazonas (IFAM), Parintins 69152-470, Brazil

**Keywords:** behavioral training, gas exchange, indirect calorimetry, Nellore, temperament

## Abstract

**Simple Summary:**

Factors such as social isolation, environmental unpredictability, and the need for physical containment are commonly present in experimental flow respirometry trials, which in turn are highly likely to induce voluntary and the autonomic fear responses in animals. These responses can introduce biases, often invisible, to study interpretations. In this study, we employed a protocol to train 30 Nellore cattle for non-ventilated and valved facial mask flow respirometry trials. The training lasted 127 days and consisted of gradually altering the animals’ environment and associating it with positive stimuli. During this period, we evaluated the pattern of evolution of the animals’ reactivity through behavioral and physiological measures. An unsupervised artificial intelligence model was employed to identify dissimilarity patterns among the animals. Overall, animals classified as less reactive (*n* = 17) presented reduced aversive behavioral responses to containment (e.g., trunk movement, attempts to remove the mask) and to the use of the valved facial mask, while those more reactive (*n* = 13) had a progressive increase in these responses. At the end of the training, less reactive animals could be contained in the chute and wear the valved facial mask for 25–30 min without apparent behavioral and physiological changes.

**Abstract:**

Training is instrumental in identifying and selecting cattle that exhibit greater cooperation with experimental conditions required in flow respirometry assays, like restraint and the use of a valved facial mask. In our study, a tailored training protocol for Nellore cattle facilitated their participation in flow respirometry assays with a valved facial mask. Over 127 days, 30 entire Nellore males, weighing 450 ± 25 kg and averaging 32 ± 2 months, underwent training from May to September 2022. The regimen involved gradually altering the animals’ environment and providing positive reinforcement, divided into three phases. Physiological and behavioral responses to containment routines and facial mask use were meticulously assessed. Principal component analyses revealed dissimilarity patterns among the animals. Animals classified as less reactive showed increased acceptance of handling, reduced reactions to weighing, and greater tolerance of the facial mask. In the final phase, the least reactive animals tolerated wearing a valved mask for extended periods without notable changes in respiratory rate. The training protocol effectively identified and selected Nellore cattle displaying enhanced cooperation with restraint and mask use during flow respirometry assays, without apparent behavioral or physiological alterations.

## 1. Introduction

Indirect calorimetry methodologies have been employed to study gas exchanges and animal bioenergetics, particularly to determine methane emission rates [1,2,3,4], one of the main greenhouse gases derived from animal production, especially ruminants [5]. In these studies, whole-body respirometric chambers [6,7,8], ventilated hoods [6,9,10], and valved facial masks [2,11,12,13] are employed. However, factors such as social isolation, environmental unpredictability, human presence, squeeze chute, and, in some cases, the use of facial apparatus are present in these methodologies and can elicit fear responses in animals. Such responses include voluntary escape and agitation reactions, as well as autonomic changes in the respiratory and cardiovascular patterns of the animals [14,15,16].

The magnitude of these changes varies according to the personality/temperament of the animals [17]. It is highly likely that individuals within the same group perceive and respond differently to stimuli presented during respirometry and/or digestibility trials [18,19], thus inducing bias in the results, a frequently overlooked aspect in studies of this nature [20]. In a group of Girolando cows, animals more reactive to stimuli presented during whole-body respirometry experiments emitted 35% more methane than less reactive ones, on average [21]. However, training animals using basic principles of associative learning can be useful in reducing undesirable responses during evaluations (e.g., agitation, escape attempts, vocalizations), as well as selecting those more cooperative [22] with containment and use of a valved facial mask, for example [2,12,13]. Valved facial masks add some resistance to the animals’ breathing, and in more agitated ones, this effect can be exacerbated, causing an undesirable physiological change such as a progressive increase in respiratory rate [23]. In a recent study, involving sixteen Morada Nova sheep submitted to a training for flow respirometry trials with a valved facial mask, 70% of the animals progressively reduced undesirable behaviors (e.g., agitation, attempts to remove the mask, and vocalizations). By the end of training, they used the valved facial mask for 40–50 min without changes in respiratory rate [24].

The selection of more cooperative animals also reduces the risks of accidents and injuries, both for the animals themselves and for observers/handlers, especially when dealing with large animals like cattle. In Brazil, Nellore cattle (Bos indicus) are predominant in beef production systems, which often end in extensive pasture areas. Representing 80% of the national cattle herd, Nellore has more than 220 million heads in Brazil, adaptability to the tropical climate and good performance response favored its expansion [25]; for this reason, we decided to use the Nellore breed in our research. Under such circumstances, these animals experience low levels of interaction with humans, except during less-frequent handling routines in the corral, which are mostly perceived as aversive [26]. Indirect calorimetry methods have been frequently employed to determine the impact of nutritional additives and ruminal fermentation manipulators on enteric methane emission rates of Nellore cattle and their crosses. However, they neglect the impacts of potential individual differences in reactions to stimuli presented during evaluations. In this study, 30 Nellore cattle underwent a training period to identify and select individuals more cooperative with the environmental conditions required in flow respirometry trials using a valved facial mask. The training protocol employed was based on gradual and regular alteration of the animals’ environment, in association with positive sensory and nutritional stimuli. Our hypothesis is that the training is adequate to select less reactive and more cooperative Nellore cattle adults to be restrained and wear a valved face mask for 20–30 min, without undesirable behavioral or physiological changes.

## 2. Materials and Methods

### 2.1. Animals, Location and Diet

The study was approved by the Ethics Committee for Animal Use of the São Paulo State University, Jaboticabal, São Paulo, Brazil, (Protocol: 1256/2022). Thirty purebred Nellore bulls (450 ± 25 kg live weight) with an average age of 32 ± 2 months in good health status were randomly selected. They were transported to the research site, at the Animal Bioclimatology Laboratory of São Paulo State University Júlio de Mesquita Filho (UNESP), Campus of Jaboticabal (21°8′ S; 48°11′ W), São Paulo, Brazil. The animals were housed in individual pens of approximately 18 m^2^ each, partially covered with galvanized tiles, fenced with wooden boards approximately 2 m high, with natural ventilation and equipped with a feeder and drinking fountain with ad libitum access. The 30 cattle remained in the pens for a total period of eight months (between May and December 2022). For training purposes, the structures used included an arena of approximately 6 m^2^, a syringe, a chute, and a squeeze chute covered with galvanized tiles to provide protection from solar radiation.

On the training days, the diet consisted of corn silage-based feed with one kilo of concentrated commercial cattle feed (based on soybean bran, wheat bran, corn grain, livestock urea, calcium carbonate, sodium chloride, dicalcium phosphate, calcium iodate, sodium selenite, cobalt sulfate, zinc sulfate).

Dry matter analyses were performed to assess the need for daily adjustments. Approximately 200 g of concentrated feed samples and approximately 350 g of samples of the silage to be provided were collected. Prior to the morning feeding, samples of leftover diet from each trough were also collected. These samples were placed in an oven, divided into triplicates and taken to an oven at 105 °C, without forced air circulation, for at least 12 h [27].

### 2.2. Experimental Routine and Training Process

The training process was performed employing the principles of habituation and conditioning to reduce fear responses [28] to the squeeze chute, human presence, and facial mask use. The total training duration was five months (between May and September 2022). Training was conducted daily (except on Saturdays and Sundays) from 8:00 am to 5:00 pm. The animals received their training diet (which was prepared individually before the start of activities) upon returning to their pens after the evaluation period in the squeeze chute. A consistent schedule and staffing levels were maintained. At the end of the training tasks, the animals received positive reinforcement [16].

The training was divided into the following stages: 1st phase: Initial habituation to the data collection site and pre-evaluation of animal reactivity; 2nd phase: Desensitization to halter use, brushing, and staying in the chute; 3rd phase: Adaptation to the use of the facial mask and the facial mask with attached valve. The classification of groups was based on the following characteristics: Group 1: Calm animals, showing the non-reactive or least reactivity. Group 2: Agitated animals, displaying low reactivity but with increased tail movement. Group 3: Reactive animals, exhibiting high reactivity.

#### 2.2.1. Phase 1: Initial Habituation to the Data Collection Site and Pre-Evaluation of Animal Reactivity (40 Days)

The adaptation period occurred 10 days after the animals arrived at the experimental facility. During this process, handlers calmly passed by the pens externally at least five times a day, providing opportunities for visual contact between the animal and the handler. This activity was carried out to acclimate the animals to the presence of the handlers [29]. To enhance the human–animal relationship, the same handlers provided a diet based on corn silage, served in individual troughs in their pens.

Afterwards, the training protocol began with all animals. This involved familiarizing the animals with the data collection site (alleyway, handling chute, and arena). Individually, each animal was led to the training areas by the same experienced trainer, who used a stick with a flag at the end to help guide the animal [30]. The animals were directed to the handling chute, then to an arena without being restrained, and finally returned to their pens. Upon returning from the activity, the animals were provided with their corn silage-based feed with one kilo of concentrated commercial cattle feed. This process was repeated for approximately 10 days.

We carry out a pre-assessment to identify animals that are difficult to handle, identifying those that have difficulty moving from the pens to the containment ramp. During this period, each animal received an individual number, marked on the right side, in the hip region, behind the flank line to facilitate individual differentiation. This number was also the same number as the stall in which that animal was housed (1 to 30).

At the beginning of phase 1 of the conditioning, the animals underwent a routine of activities after initial habituation to the collection site, which included leading the animal from its pen to the handling chute and evaluating the animals in the handling chute. Reactivity measures to the handling chute were collected through direct observation of behavior using a Focal sampling route, where the observer focused on a specific, identified animal [31]. Our study employed 5 visual scores: tail score adapted from [32,33,34], movement in the chute adapted from [34], movement on the chute adapted from [32,33,34], balance score adapted from [35], and exit score adapted from [36], which were assigned within four seconds after closing the entry and exit gates of the chute. Each evaluated variable received a score from 1 to 5.

**Figure 1:**
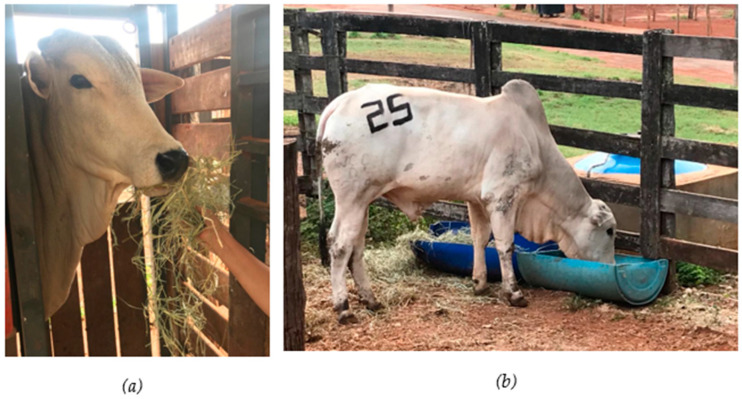
The animals in training received positive reinforcement at the end of the evaluation; (**a**) squeeze chute and (**b**) in the arena.

During this phase, which lasted approximately 20 days, notes were made about reactivity associated with the time spent in the chute. This time was progressively increased (in the first few days, the animals only passed through the chute; later, the time of stay was increased to 1 min., 2 min., *n* min.). After five days, with a stay time of 2 min in the chute, brushing of the dorsolumbar region of the animals with a soft-bristled brush was added to the protocol. The animals in training received positive reinforcement at the end of the evaluation in the squeeze chute (Figure 1a) and in the arena (Figure 1b).

#### 2.2.2. Phase 2: Desensitization to Halter Use, Brushing, and Squeeze Chute Permanence (46 Days)

At the end of June 2022, desensitization began along with brushing the animals, both on the body and on the face, for halter with chain use. This phase lasted approximately 10 days and was conducted to reduce sensitivity before putting the halter on the animals. The animals were also brushed gradually from the back to the rump with a soft brush [37]. To desensitize the head area, a flag was used, made from a pole padded with a feed bag.

The movements were gentle, with massages aimed at touching the parts of the animal’s head that would come into contact with the halter [38]. The animals that responded best to the desensitization process with the flag and body brushing were haltered with a rope halter in July 2022. Then, the selected animals began training while also wearing an halter.

#### 2.2.3. Phase 3: Adaptation to the Use of the Facial Mask and the Facial Mask with Attached Valve (41 Days)

**Figure 2:**
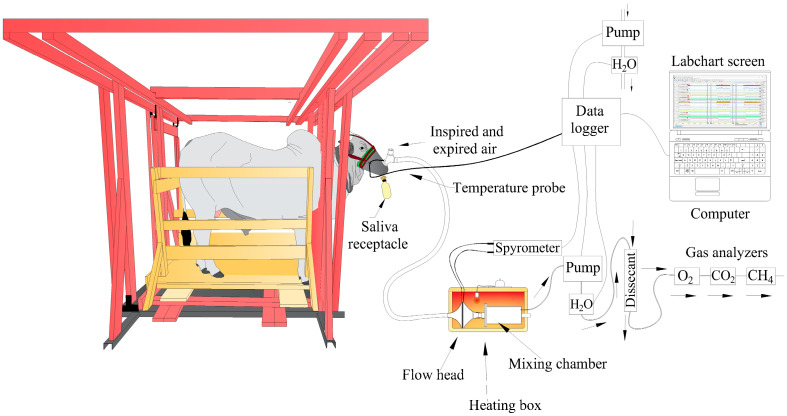
Design of the system for thermal equilibrium evaluation, adapted from [39].

The animals that responded best to the use of halters were subjected to a new protocol in August 2022, now with the use of the facial mask without the attached valve. The conditioning period for mask use was approximately 20 days, with a progressive increase in time spent in the squeeze chute [29]. To assess the mask-wearing behavior, head movements (upward, downward, sideways, whether tossing the facial mask or not) and neck movements (forward, upward, downward, or backward, pushing the neck against the neck opening) were observed, providing scores from 1 to 5 (adapted from [34]). A score of 1 was considered as little or no movement of the head and neck in an attempt to remove the mask, while a score of 5 indicated very vigorous movements, with high frequency in the animal’s attempt to remove the facial mask. After evaluating the animals’ responses, a valve was attached to the facial mask, and the animals were trained and evaluated for an additional 5 days. In October 2022, the animals that were less reactive to the conditioning with the facial mask underwent training in the respirometry system (approximately 5 days), meaning that tests were conducted using the complete facial mask in the system (Figure 2).

**Figure 3:**
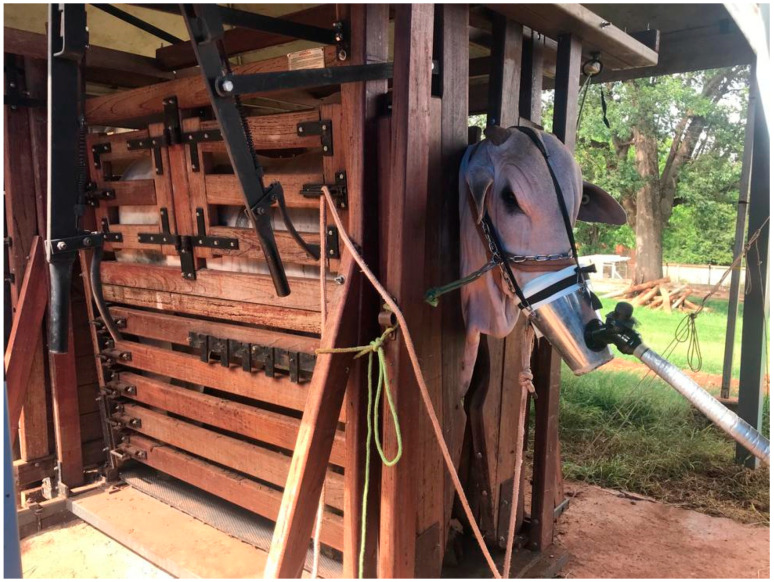
Bull wearing face mask connected to system.

Based on the preliminary results, the best animals were selected to undergo the use of the indirect calorimetry system to measure respiratory gases without forced air circulation using a valved facial mask (Figure 3).

The facial mask was positioned carefully to make sure that the volume of the ventilated dead space (Vd) was as close to zero as possible, because it affects the true concentration of the expired gases [40]. The designed masks were built with the lowest possible Vd (Vd→0); hence, the ratio (K) of the respiratory tidal volume (VT) to Vd (K= Vd/VT) was as large as possible. In this case, the geometry of facial mask that reduced the Vd was an ellipsoidal shape. We were also careful to make ensure that all of the expired air flowed through the mask and into the valves without any leak. It was sealed around the muzzle of the animal using rubber sheet (a dental product) to avoid leakage of air [41].

### 2.3. Statistical Analysis

At the end of each phase, principal component analysis was performed to group the animals into one of the three groups (calm, agitated, and reactive animal). Five score measurements were carried out (scores on a scale of 1 to 5), behavioral responses, measured immediately after putting on the mask and then every ten minutes, at regular intervals, until completing one hour of observation. At the end of conditioning, a preliminary assessment of respiratory rate was carried out to check whether the use of the face mask was affecting respiratory rate (RR, breaths per minute). Ten animals were evaluated per day for fifteen consecutive days.

The RR data were analyzed according to the following statistical model:

$$Y_{\mathrm{ijkLm}}=\mu +D_{i}+{A_{j}(D)}_{i}+R_{k}+T_{L}+{\left(DT\right)}_{\mathrm{iL}}+T_{L\;}x+e_{\mathrm{ijkLm}}$$

In the model, *Y*_ijkLm_ represents the *m*-th observation of the respiratory rate, where *D* is the fixed effect of the *i*-th collection day (*i* = 1, …, 15), *A* is the fixed effect of the *j*-th animal within the *i*-th collection day (if *j* = 1, …, 10 then *i* = 1, 4, 7, 10, and 13; if *j* = 11, …, 20 then *i* = 2, 5, 8, 11, and 14; and if *j* = 21, …, 30 then *i* = 3, 6, 9, 12, and 15), *R* is the fixed effect of the *k*-th repetition (*k* = 1, …, 5), T is the fixed effect of the *L*-th time interval (*L* = 0, 10, 20, 30, 40, 50, and 60 min), *DT* is the effect of the interaction between the *i*-th collection day and the *L*-th time interval, T*A(*D*) is the effect of the interaction between the *L*-th time interval and the *j*-th animal within the *i*-th collection day, *e*_ijkLm_ is the random error, and *µ* is the parametric mean.

The principal component analysis as a clustering analysis [41,42] was used to group different animal temperaments regarding the use of the facial mask (calm animals: showing the non-reactive or least reactivity; agitated animals: displaying low reactivity but with increased tail movement; and reactive animals: exhibiting high reactivity). The PRINCOMP procedure of SAS was used for principal component analysis. To classify animals according to their temperament regarding the use of the facial mask, a reactivity index was developed in ascending order. The variables tail movement (TM), movement to the chute (MTC), movement in the chute (MIC), balance score (BAL), and chute exit (CE) were summarized into a single variable through principal component analysis, following the procedures described in [42] and applied by [43].Taking the correlation matrix of the response variables, RYY, we have $e_{1}^{\prime }=\left[e_{11}\;\ldots {\;e}_{17}\right]$ as the eigenvector associated with its largest eigenvalue (λ1), and whose elements correspond to TM, MTC, MIC, BAL, and CE, respectively. Then, the first principal component was:

$$y_{1}=e_{11}TM+e_{12}MTC+e_{13}MIC+e_{14}BAL$$

This first component was one index tested, but several other indices were constructed, following the Value Analysis of Membership Function cited by [44,45]. After testing these indices, the chosen one was used to rank the animals from least reactive to most reactive to the use of the facial mask, with the first 12 selected for use in the gas exchange assessments in the respiratory system.

## 3. Results

### 3.1. Phase 1: Initial Habituation to the Data Collection Site and Pre-Evaluation of Animal Reactivity

**Figure 4:**
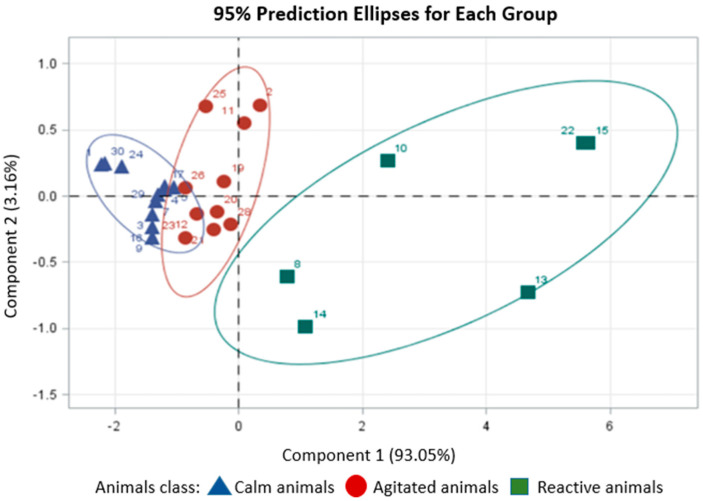
Bi-plot between the first two principal components based on five reactivity responses of the animals (tail movement, displacement in the chute, movement in the chute, balance score and exit score) in the squeeze chute.

The patterns of dissimilarity among the animals were evaluated through multivariate analysis of their reactivity responses during Phase 1 of the training. According to the dissimilarity patterns from the principal component analysis, the animals were divided into the following groups (Figure 4):

Group 1 (1, 3, 4, 5, 7, 9, 16, 17, 24, 29, and 30): These animals had the lowest reactivity scores in the containment chute. Group 2 (2, 11, 12, 19, 20, 21, 23, 25, 26, and 28): these animals were characterized as agitated. Group 3 (8, 10, 13, 14, 15, and 22): These animals did not reduce their reaction to the handler’s presence and were grouped as highly reactive.

The animals in group 3 did not accept the necessary handling to leave the pen and pass through the trunk, which made handling impossible due to their highly reactive temperament. These animals were removed from the training routines.

### 3.2. Phase 2: Desensitization to the Use of the Halter and Permanence in the Squeeze Chute

**Figure 5:**
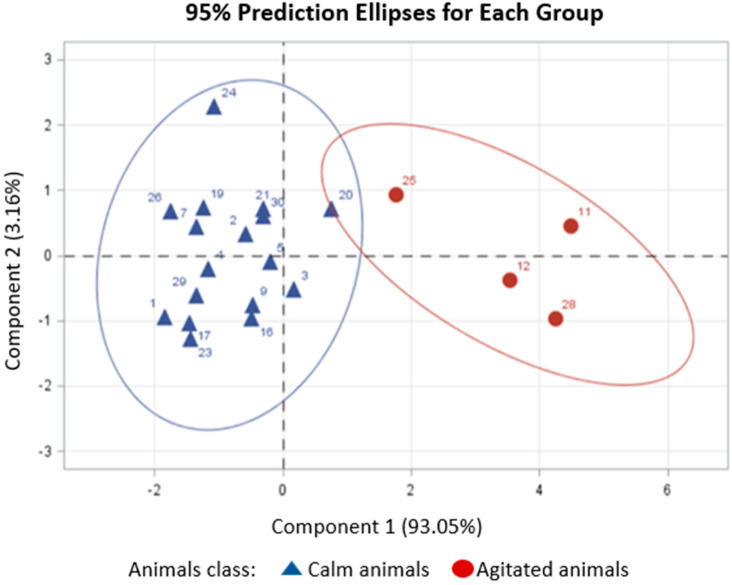
The bi-plot of phase 2 between the first two components of the principal component analysis is based on five reactivity responses of the animals (tail movement, movement in the chute, movement in the chute, balance score, and exit score) in the squeeze chute.

At the end of each conditioning phase, it was possible to group the animals based on the evaluations conducted in the squeeze chute. After the first 40 days of training, in phase 1, animals classified as calm and agitated were selected to participate in phase 2. After the desensitization period to the use of the halter using the flag and brushing the body of the animal from the back region to the rump, group 1 (1, 2, 3, 4, 5, 7, 9, 16, 17, 19, 20, 21, 23, 24, 26, 29, and 30) were animals that responded best to the second phase, being classified as calm animals (Figure 5).

### 3.3. Phase 3: Adaptation to the Use of the Face Mask and the Face Mask with Attached Valve

**Figure 6:**
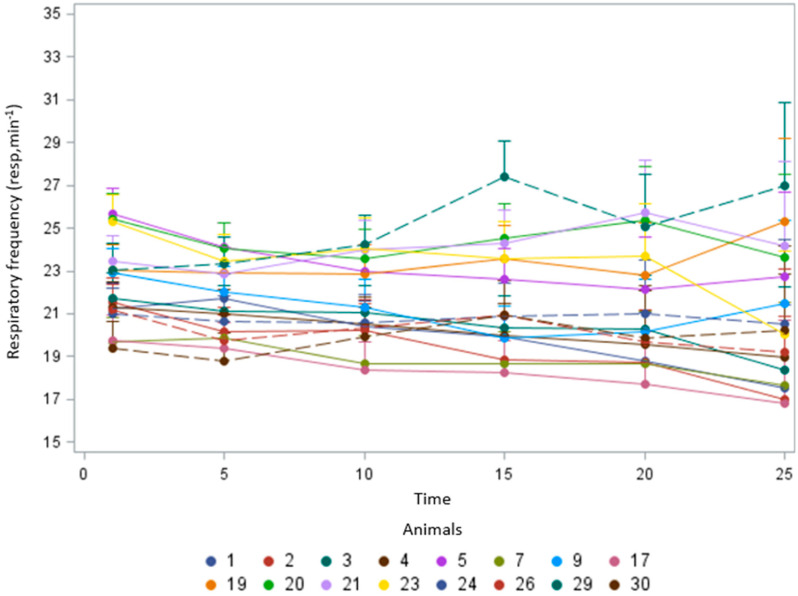
Respiratory frequency responses over time of the selected animals subjected to the face mask test and permanence in the squeeze chute.

Group 1 (calm), represented by animals 1, 2, 3, 4, 5, 7, 9, 17, 19, 20, 21, 23, 24, 26, 29, 30, responded less reactively to the use of the halter, face mask, and staying in the squeeze chute. In phase 3, these animals underwent evaluations of respiratory frequency, and responses over time were obtained. During the evaluation period, variations in the animals’ respiratory frequency were observed over time intervals of 5, 10, 15, 20, and 25 min (Figure 6). Among the animals selected after wearing the face mask, animal 3 reached respiratory frequency values above 27 breaths per minute at the 15 min mark. However, at 20 min, there was a reduction to 25 breaths per minute, with a slight increase at 25 min with the use of the face mask.

To form the experimental groups for the collection of respiratory gases, 12 animals (2, 4, 5, 7, 17, 19, 20, 21, 23, 24, 26, and 30) were selected. Out of the total of 30 animals received for the execution of the experimental protocol, 16 animals participated in the training phase. Table 1 presents the evolution of the animals’ behavior during the training period (phases 1, 2, and 3) after evaluation to which they were subjected and TM (Tail Movement), MTC (Movement to the Chute), MIC (Movement in the chute), BAL (balance score), and CE (Chute Exit).

The scores applied (1 to 5) within each assessment carried out, TM (Tail Movement), MTC (Movement to the Chute), MIC (Movement in the chute), BAL (balance score), and CE (Chute Exit), in phases 1, 2 and 3, resulted in a gradual reduction. For the variables tail movement, movement to the chute, and movement in the chute, 63.50% of the animals were evaluated with a score of 1 and 0.38% with a score of 5. As the training progressed, in phase 2, a greater number of animals were observed (78.75%) evaluated with score 1 and no animal evaluated with score 5. For phase 3, it was observed that for the variables TM, MTC, MIC, and BAL, all animals (100%) were evaluated with score 1. The variable CE reduced the percentage of animals evaluated with score 5 in phase 1 (1.16%) and phase 2 (0.37%), and almost 60% of the animals were evaluated with score 1, resulting in a significant reduction in the reactivity assessment of animals that previously had a score of 5.

At the end of the pre-established confinement period, the animals were slaughtered at a commercial abattoir, following the norms and guidelines of the slaughterhouse. The animals were slaughtered to sell the meat on the domestic market.

## 4. Discussion

Based on the results of this study, the hypothesis that the reactivity of Nellore cattle can be reduced for use in flow respirometry tests can be confirmed by following a stipulated training protocol. In this sense, to avoid or nullify discrepant changes in the results, we used criteria for selecting the best animals using habituation and operant conditioning techniques, resulting in the selection of 12 animals for participation in the experimental phase.

To obtain positive results in the conditioning of the animals, it was necessary to follow a set of procedures that include trained personnel, routine (management), and an environment conducive to the process. Due to prior planning, on the stipulated training days (approximately 10 to 15 consecutive days), generally at the same time, the same team carried out the established activities. The use of trained personnel is associated with better animal behavior, which includes reduced reactivity and stress, making positive human–animal interactions a practical and effective strategy [46]. A favorable environment is also an important factor for cattle learning. Conditioning (or associative learning) occurs when animals establish connections between certain situations (places, people, etc.) and sensations [47]. Based on these three pillars (trained personnel, management, and environment), the training protocol for confined cattle was described as follows:

### 4.1. Phase 1: Initial Habituation to the Data Collection Site and Pre-Assessment of the Animals’ Reactivity

For a favorable environment, the initial habituation of the animals to the location and activities is the first step in the process that must be considered. Changes in the environment and activities should follow a gradual procedure, respecting the stipulated time for each task. So, changing the environment or activities causes a disruption in the social activities of cattle, making it difficult to maintain individual space and breaking the balance in the dominance hierarchy [47]. Considering that cattle are gregarious animals, respecting their individual space helps them remain in harmony. In contrast, deviation from this process, especially in intensified farming systems, leads to fight-or-flight attempts, causing stress [18]. Understanding the potential implications during management can prevent avoidant responses from the animals, which cause physiological changes and contribute to longer activity (training) times and the risk of accidents.

The adaptation period of the animal to its new environment, which can be associated with positive stimuli, can reduce stress impacts [48]. Positive reinforcement is a technique that successfully achieves satisfactory results in cattle management, as found by Moura et al. [2]. Besides being a significant contribution to animal welfare, using positive reinforcement during training [49] can lead to associative learning with the proposed activity. Frequent handling over time can establish a positive relationship with human presence and reduce the animals’ fear, aiding the conditioning process [50]. Incorporating rational handling techniques is essential for maintaining behavioral responses in the human–animal relationship, especially when animals spend extended periods in research. Employing habituation techniques, the animal’s motivation combined with the learning process can yield more satisfactory training results. Nawroth et al. [48] mention in their study on the cognition of livestock species that cattle’s ability to communicate with humans can impact management practices by improving handling routines.

In this regard, it was possible to observe that out of the 17 animals, there was progress in the training process during phase 1, as demonstrated by the use of principal component analysis. This analysis separated animals that responded positively to the conditioning process employing positive reinforcement before and after evaluations (Figure 2). In other words, in intensive handling yards, the practice of habituation and conditioning of animals assists in management activities [28]. Paranhos et al. [51] described that cattle have good memory and respond well to routines. They are capable of distinguishing the people involved in interactions and exhibit specific reactions to each of them, depending on the type of experience lived, characterizing associative learning of the operant conditioning type.

### 4.2. Phase 2: Desensitization to the Use of the Halter and Permanence in the Squeeze Chute

The halter is an important tool for keeping both the animal and its handlers safe. Quintiliano and Paranhos da Costa [28] describe that cattle have a high learning capacity, and procedures of habituation and operant conditioning are beneficial when handling without aversive stimuli. In this case, the use of palatable food reinforcement for animals, as well as employing differentiated time in conditioning for more reactive animals, often becomes necessary. In this sense, it was possible to observe in our results that animals classified as calm responded progressively to the handling performed, demonstrating maintained calm of behavioral responses to the squeeze chute. On the other hand, animals classified as agitated and reactive exhibited greater reactive responses at the beginning of the process, but over time, these responses decreased and remained constant. Thus, the use of differentiated time for the specificity of each animal was positive throughout the process.

The human–animal relationship may not always yield the expected responses for all animals, even if the handler has always been the same, using the same methods, attire, and tone of voice. Considering that there is abundant evidence indicating that individual differences in temperament are genetically influenced, studying the environmental contributions to temperament is equally important [17]. In this regard, it was possible to observe that even when following the same management standard for all animals, their reactive responses differed. In other words, despite providing the same environment, trainers, and positive reinforcement in equal measure, some animals responded positively to the protocol, while others reacted more sensitively to the handling, increasing their reactivity when they remained in the handling trunk with more vigorous body and head movements.

Muller and Keyserlingk [52], when measuring the exit velocity in Bos taurus indicus animals, also found a slight increase between the second and third evaluations and suggested that repeated handling of the animals led to an increase in their fear. Repeated handling can habituate animals to certain activities; however, in some animals, the repetition of a certain activity can develop sensitivity reactions, increasing their state of fear, which occurred in some animals in our study. It was observed that some animals may not habituate to handling, maintaining a high reactivity index that may increase as training progresses. Silveira, Fischer, and Soares [53], when evaluating the effect of the ethological group (crossbred, European, and Indicus animals represented by the Nellore breed), found a higher percentage of Indicus animals, followed by crossbred and European animals with higher behavioral scores (3 and 4) than European animals, respectively, 60%, 33%, and 5.5% of the animals. They concluded that the ethological group significantly influenced the temperament of extensively raised animals, where Indicus and crossbred animals exhibited higher reactivity than European animals. Even with attention focused on the animal, the environment, and the trainers, this genetic characteristic of Indicus animals may have contributed to the longer training time for animals coming from extensive production systems, partly due to the maturity condition of the animals and their weight, averaging nearly 500 kg, implying greater difficulty for the animals to accept handling.

Based on the results, 16 animals were selected for phase 3, which responded better to the use of rope and halters, as well as permanence in the squeeze chute.

### 4.3. Phase 3: Adaptation to the Use of the Facial Mask and Permanence in the Squeeze Chute

Research on reactivity in farming systems demonstrates that the fewer the interactions with humans, the more absent the human–animal interaction, the greater the attempts by the animal to respond to the proximity actions of the handler [26]. Our results showed that with increased human–animal interaction, 17 animals progressed to phase 3 (1, 2, 3, 4, 5, 7, 9, 17, 19, 20, 21, 23, 24, 26, 29, 30). However, animal 16 was excluded from the group due to illness recovery, resulting in a total of 16 selected animals trained with the facial mask and the facial mask with the attached valve. With increased exposure time of the animals to training, there was a reduction in reactive fear and stress characteristics in the presence of the handler, contributing to the reliability of gas readings by indirect calorimetry with the aid of a non-vented facial mask.

In general, the results presented in Figure 3 and Table 1 show a slight stability in the reactivity of the animals in the handling chute over the course of days. It was observed that when employing the training protocol, not all animals were ready to participate in the experimental period, indicating individual differences in the temperament of each animal. Genetic factors influencing temperament are intrinsic to the animal [18]. It can be noted that the animals’ responses to training did not all occur simultaneously; some animals took longer to reduce their reactivity to handling, as described by Grandin and Deesing [17], where it is possible to reduce stress levels in excitable animals and thus facilitate handling, but it is important that animal training occurs very slowly. In other words, over time, it is possible to modulate using learning principles. This is consistent with the experience of individuals involved in animal husbandry, who describe differences between animals on an individual basis in their behavioral response to challenging or alarming situations [54]. The positive or sensitive responses of animals to a handling process are intrinsic to them, as observed among the animals in group 3 (Figure 2).

The presence of valves in the facial mask in phase 3 may have increased resistance during animal breathing, causing resistance to use and leading to slight changes at the beginning of the time after putting on the mask. Nevertheless, in the evaluation of respiratory rate and frequency, it is possible to verify that there was little variation in response over time, observing how each individual responded to the protocol and demonstrating fewer responses to the use of the facial mask and remaining in the squeeze chute. Haskell, Simm, and Turner [54] describe that when there is a reduced response to stimuli, there is habituation or conditioning of the animals. After 80 days of training, it was recorded and analyzed that the responses of the animals that best accepted the conditioning protocol remained within the group of animals evaluated as calm, making it possible to select the least reactive animals without interpolating to other measures.

## 5. Conclusions

(1) The training protocol, it was possible to select less reactive and more cooperative Nellore bulls in respirometry flow trials using a non-ventilated facial mask.
(2) It was possible to verify that some animals become accustomed to the training protocol while others become sensitized.
(3) To obtain safe and accurate measurements, the use of the training protocol was fundamental in flow respirometry assays.

## Figures and Tables

**Table 1:** Evaluation of Nellore cattle balance over the phases.

Score (%/Frequency)						
		1	2	3	4	5
TM	Phase 1	63.50	22.81	9.89	3.42	0.38
	Phase 2	78.75	9.89	8.79	2.56	
	Phase 3	100				
MTC	Phase 1	31.56	43.73	18.65	3.80	2.66
	Phase 2	64.29	26.59	5.95	3.17	
	Phase 3	100				
MIC	Phase 1	25.10	38.78	28.90	4.18	3.04
	Phase 2	54.37	32.14	9.92	3.57	
	Phase 3	100				
BAL	Phase 1	15.21	40.68	30.80	10.27	3.04
	Phase 2	47.22	33.33	5.16		
	Phase 3	100				
CE	Phase 1	36.43	47.67	10.47	4.26	1.16
	Phase 2	42.49	41.76	14.29	1.10	0.37
	Phase 3	59.39	32.08	8.53		

## Data Availability

The data presented in this study are available upon request from the corresponding author.
